# Enhancing the clinical relevance of haemorrhage prediction models in trauma

**DOI:** 10.1186/s40779-023-00476-6

**Published:** 2023-09-20

**Authors:** Sankalp Tandle, Jared M. Wohlgemut, Max E. R. Marsden, Erhan Pisirir, Evangelia Kyrimi, Rebecca S. Stoner, William Marsh, Zane B. Perkins, Nigel R. M. Tai

**Affiliations:** 1 Centre for Trauma Sciences, Blizard Institute, Queen Mary University of London, London, E1 2AT UK; 2 The Royal London Hospital, Barts Health NHS Trust, London, E1 1FR UK; 3 Academic Department of Military Surgery and Trauma, Research and Clinical Innovation, The Royal Centre for Defence Medicine, Birmingham, B15 2WB UK; 4 Department of Electronic Engineering and Computer Science, Queen Mary University of London, London, E1 4NS UK

**Keywords:** Trauma, Injury, Blood transfusion, Massive transfusion, Prediction, Artificial intelligence, Machine learning

We read with interest the recent systematic review “Artificial intelligence and machine learning for hemorrhagic trauma care” by Peng et al. [1], which evaluated literature on machine learning (ML) in the management of traumatic haemorrhage. We thank the authors for their contribution to the role of ML in trauma.

Prediction of relevant patient outcomes may inform clinicians about the severity of injury and guide their decisions on further treatment. However, many prediction algorithms developed for traumatic haemorrhage, including those which utilise ML, predict binary outcomes built on training datasets where prior clinical decisions are modelled. Such an approach can be problematic. This commentary highlights these issues, and offers an alternative predictive approach.

The decision to transfuse an injured patient rests with the treating clinician. This decision relies on the clinician’s assessment of the patient’s requirement for blood products, which will be based upon the integration of relevant patient information with the clinician’s own experience. Transfusion is often a time-critical step in the treatment of haemorrhaging trauma patients and there may be uncertainty in decision-making. An incorrect choice exposes patients who are over-treated to the risks of a needless transfusion, or the consequences of inadequate resuscitation to the under-treated. Mismatching of patient to therapy risks waste of precious blood stocks—especially when resources are constrained by mass casualty events or the austere setting. Accordingly, several scoring systems, of variable complexity, exist to aid decision-making. Many of these scoring systems were identified in a recent narrative review, including the Assessment of Blood Consumption score, the Trauma Associated Severe Hemorrhage score, and the McLaughlin score for combat casualties [2]. However, such scores predict the need for massive transfusion: a dichotomous outcome that is problematic for several reasons, and clinical uptake has been limited.

Firstly, the criteria denoting massive transfusion (patients receiving 10 units of packed red blood cells) is arbitrary and there is little to suggest that patients predicted to require 9 or 11 units differ in their physiology, requirement or outcomes. Dichotomous predictions ignore the distinctive patient groups in the higher transfusion range; information that is especially useful in military practice when expeditious but carefully balanced decisions around limits of care must be made. For this reason, prediction of nominal categories may be of better utility as it improves granularity of the outcome. Knowing a patient is likely to consume 20, 30 or 50 units of blood (as opposed to more than 10 units) can inform decision-making on triaging patients to facilities with sufficient blood stocks. Additionally, it allows blood banks in these facilities to mobilise blood products and reduce ‘door-to-intervention time’. Ideally, the prediction of absolute values would provide the greatest utility, though there are recognised methodological frictions (largely concerned with the diminishing number of cases per value from which the ML can be trained).

Secondly, predicting whether a patient falls into an arbitrarily defined group, based on historic clinical choices embedded within ML training data, does not offer the same value to the decision-maker as prediction of underlying requirement—that is, the true patient state. In the context of traumatic haemorrhage, models predicting transfusion requirements based on training data risk propagating clinical decisions of possible under- or over-transfusion. One of the biggest issues in artificial intelligence research currently is the problem of unconscious biases being trained into models that perpetuate those biases. Conversely, models that predict clinical need for transfusion are less likely to be affected by this issue, and will be less affected by trends in practice such as the move toward whole blood transfusion, or changes in component therapy.

Thirdly, both of these factors may compound and serve to exacerbate survivorship bias [3]. Framing the learning of ML models around whether a patient meets a threshold (such as massive transfusion) as learnt from historic clinical judgements encoded in the training data risks excluding patients with the requirement for transfusion who do not meet the massive transfusion threshold as they do not survive long enough to receive it. Whilst identification and accounting for severely injured patients who die before they meet such a transfusion threshold can reduce bias, designing models that are based around requirement, and which provide finer calibrations of that requirement, will also reduce error whilst improving utility.

Fourthly, the timeline of many existing prediction outputs does not provide clinically useful information for decision-making about resuscitation intensity. Predicting whether a patient will require 10 units of packed red blood cells within 24 hours, is not only an arbitrary volume cut-off, it is also an arbitrary time cut-off. There has been a shift towards sooner endpoints for trials investigating traumatic haemorrhage, due to evidence that most patients who die from haemorrhagic shock succumb within 6 hours from injury [4]. Thus, there is good reason to predict resuscitation within 6 hours, as the window for treatment with blood products is within this timeframe. Prediction at this early timepoint must draw from information available immediately after injury, including aggregate markers of physiology, injury burden and mechanism.

While it is desirable to have ML models that predict multiple clinically useful endpoints, the first steps are to determine whether: the model can predict patient-centred, measurable endpoints better than existing methods; these results are reproducible, explainable and understood by users; the system is usable and useful in the field; and most importantly whether the prediction system leads to improved patient outcomes.

Future efforts at developing ML haemorrhage prediction models in trauma should aim to avoid dichotomous thresholds, predict transfusion requirements, and focus on the first few hours after injury.

## Data Availability

Not applicable.

## Abbreviation

ML: Machine learning

## References

[1] Peng HT, Siddiqui MM, Rhind SG, Zhang J, Teodoro da Luz L, Beckett A (2023). Artificial intelligence and machine learning for hemorrhagic trauma care. Mil Med Res.

[2] El-Menyar A, Mekkodathil A, Abdelrahman H, Latifi R, Galwankar S, Al-Thani H (2019). Review of existing scoring systems for massive blood transfusion in trauma patients: where do we stand?. Shock.

[3] Ho AM, Zamora JE, Holcomb JB, Ng CS, Karmakar MK, Dion PW (2015). The many faces of survivor bias in observational studies on trauma resuscitation requiring massive transfusion. Ann Emerg Med.

[4] Holcomb JB, Moore EE, Sperry JL, Jansen JO, Schreiber MA, Del Junco DJ (2021). Evidence-based and clinically relevant outcomes for hemorrhage control trauma trials. Ann Surg.

